# The Effect of Host-Plant Phylogenetic Isolation on Species Richness, Composition and Specialization of Insect Herbivores: A Comparison between Native and Exotic Hosts

**DOI:** 10.1371/journal.pone.0138031

**Published:** 2015-09-17

**Authors:** Julio Miguel Grandez-Rios, Leonardo Lima Bergamini, Walter Santos de Araújo, Fabricio Villalobos, Mário Almeida-Neto

**Affiliations:** 1 Programa de Pós-Graduação em Ecologia e Evolução, Instituto de Ciências Biológicas, Universidade Federal de Goiás, Goiânia, GO, Brazil; 2 Departamento de Ecologia, Instituto de Ciências Biológicas, Universidade Federal de Goiás, Goiânia, GO, Brazil; Helmholtz Centre for Environmental Research (UFZ), GERMANY

## Abstract

Understanding the drivers of plant-insect interactions is still a key issue in terrestrial ecology. Here, we used 30 well-defined plant-herbivore assemblages to assess the effects of host plant phylogenetic isolation and origin (native vs. exotic) on the species richness, composition and specialization of the insect herbivore fauna on co-occurring plant species. We also tested for differences in such effects between assemblages composed exclusively of exophagous and endophagous herbivores. We found a consistent negative effect of the phylogenetic isolation of host plants on the richness, similarity and specialization of their insect herbivore faunas. Notably, except for Jaccard dissimilarity, the effect of phylogenetic isolation on the insect herbivore faunas did not vary between native and exotic plants. Our findings show that the phylogenetic isolation of host plants is a key factor that influences the richness, composition and specialization of their local herbivore faunas, regardless of the host plant origin.

## Introduction

Ecologists have long been interested in why the species richness and composition of herbivores vary so much among co-occurring plant species [1–4]. Several empirical studies have shown that both ecological and evolutionary features of host plants influence the diversity of their insect herbivores [5–8]. The major feature that determines which herbivores can consume a given host plant species is its set of defensive barriers [9–10]. Because phylogenetically related plant species are more likely to share similar physical, chemical, and phenological characteristics than phylogenetically distant plant species [11], most herbivorous insect species are better able to colonize novel host plants when these are more closely related to their original host plants [12–14]. A consequence of such constraints is that phylogenetically isolated host plants tend to be consumed by fewer species of insect herbivores [15–17]. A shared evolutionary history among plants can also influence the diversity of plant-herbivore interactions, particularly when the host plants that evolved together with herbivores are compared with plants of alien origin. In this context, an unsolved question is how phylogenetic isolation among native and exotic host plants affects the species richness, composition and specialization of insect herbivores.

Exotic plant species are often consumed by fewer insect herbivore species than native, sympatric plants [18–22]. The chemical and structural features that distinguish exotics from native plant species [23–24] and exotic-related factors such as time since introduction [25], range size, and architectural complexity [17], are the major factors that have been tested to explain the lower herbivorous insect richness on exotic plants. Because differences in plant traits tend to be more pronounced in exotic than to native plant species [24], it can be expected that if an exotic and a native species have the same phylogenetic distance in relation to other plants in a community, a lower herbivore species richness should be observed in the exotic species.

The phylogenetic isolation of host plants can also have an impact on the specialization of their insect herbivores [14]. Such insects have evolved specialized adaptations to circumvent or detoxify plant defenses through a combination of morphological, physiological, and behavioral adaptations [26]. In addition, given the wide range of plant species available, insects have also developed adaptations to recognize their specific hosts and to discriminate the relative quality of different host species. Thus, phylogenetic isolation among host plants might lead to a greater dissimilarity of herbivores on the host plant species that are more phylogenetically isolated from the rest of the plant community [27]. In fact, several empirical studies have shown that most insect herbivore species interact with a limited number of closely related host species [28–31]. Therefore, specialist herbivores that are adapted to the physical and chemical defenses of phylogenetically isolated host plants in the community are predicted to be less likely to colonize other plant species. In the case of exotic host plant species, some studies have shown that these hosts are commonly used by a subset of generalist insect herbivores that also consume the native plants [20]. Therefore, if the effect of phylogenetic isolation is accounted for, insect herbivores of exotic host plants should be more similar and less specialized than those on native host plant species.

Variation in the richness, composition and specialization of insect herbivores on native and exotic plant species can also largely depend on the guilds of herbivores under investigation [18,25]. Insects that feed internally on plant tissues (i.e., endophagous herbivores) tend to be more specialized on their host plant species than herbivores that feed externally (i.e., exophages) [32–34]. Endophagous insects, such as gall-makers [34], leaf-miners [35] and flower-head insects [20] are examples of highly specialized herbivore guilds. Some studies have shown that the phylogenetic diversity of host plants affects the species richness of endophages [36] and that these insects are rarely found (or absent) on exotic host plants in their introduced range [37–39]. In this context, the effects of plant phylogenetic isolation and plant origin (native vs. exotics) are expected to be more pronounced on endophagous insect assemblages than in assemblages of exophagous insects.

In the present study, we compared the effects of phylogenetic isolation and plant origin (native vs. exotic) on the species richness, composition and specialization of the insect herbivore faunas using co-occurring host plant species. In addition, we evaluated whether the effects of phylogenetic isolation and plant origin are more accentuated in assemblages that are composed exclusively of endophagous herbivore insects than in those composed exclusively of exophagous insects. More specifically, we tested the following predictions: (1) plant species that exhibit a high phylogenetic isolation within a community host a poorer, more dissimilar and less specialized set of insect herbivores than plant species with a low phylogenetic isolation; (2) the effect of phylogenetic isolation on the species richness, dissimilarity and specialization of herbivore faunas will be more pronounced on exotic host plant species than on native host plant species; and (3) the effects of phylogenetic isolation and plant origin on the richness, dissimilarity and specialization of herbivore species in assemblages composed exclusively of endophages, will be higher than those composed of exophages.

## Methods

### Data compilation

**Fig 1:**
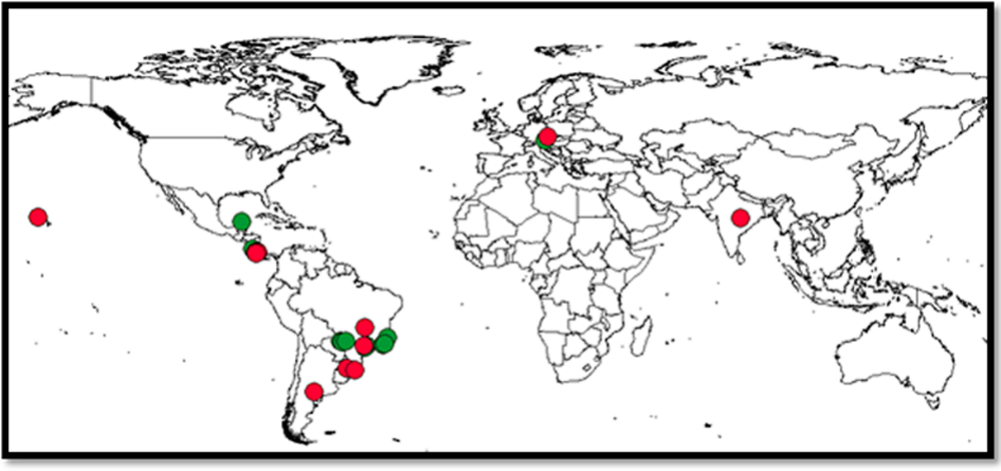
Global distribution of the 30 plant-herbivore assemblages used in this study. Green dots represent plant-insect assemblages that are composed of endophagous herbivores and red dots represent those assemblages composed of exophagous herbivores.

We carried out a comprehensive literature search for studies reporting plant-herbivore assemblages in the SciVerse Scopus, Portal Capes, and Google Scholar databases between 2011 and 2013, using the following combinations of keywords: (plant*) AND (herbivor*) AND (network* OR interaction* OR web*) AND (survey* OR list*). Only those studies with plant-herbivore assemblages that met the following criteria were included in our study: 1) at least five plant and five insect species, totaling at least 10 species for each local species list; 2) the presence of both native and exotic plant species; 3) the occurrence of at least three exotic plant species; 4) an indication that all plants could potentially be consumed by any herbivorous insect in the list (i.e., no spatial mismatch). Overall, 30 local plant-herbivore assemblages were selected from 21 studies (S1 Table). The latitudinal distribution of the plant-herbivore assemblages ranged from 49.3°N to 37.5°S (Fig 1), and their altitudes ranged from 9 to 1,430 m.a.s.l. (S2 Table).

### Plant origin and herbivore feeding mode

We categorized the origin of the host plant species as natives (naturally occurring in the region) or exotics (occurring via introduction or invasion) for each local plant-herbivore assemblage. First, we checked the synonymy using The Plant List database (www.theplantlist.org). Then, we accessed the plant databases available for each country from which samples were drawn (S3 Table). We also used additional information, such as the Invasive Species Specialist Group (www.issg.org), the Missouri Botanical Garden (www.missouribotanicalgarden.org), and Global Species (www.globalspecies.org) databases to refine our categorization of each host-plant species where they were sampled. Plant species that were determined only at the genus level (i.e., species identified as “sp.”), were categorized as exotic or native to each country according to the status of the genus. Unidentified plant species, which could not be placed in any category, were excluded from the analyses. The excluded plants represented 13% of the 662 plant species (native, exotic, and unknown) recorded in the plant-herbivore assemblages (mean = 8.2 removed species). We assumed that all assemblages were composed predominantly of native insects because native plant species were more prevalent than exotic plants in the selected plant-herbivore assemblages. In addition, the regional pools of native herbivorous insects were certainly much larger than those of exotic herbivorous insects.

Each plant-herbivore assemblage was then classified as consisting of endophagous or exophagous insect species, i.e., we separately described plant-herbivore assemblages by including all insect herbivores, or only endophagous or only exophagous insects. Thus, we obtained three lists of plant-herbivore assemblages, depending on the type of insect herbivore considered. We categorized those herbivores that feed concealed within plant tissues (e.g., gall makers, leaf miners) as endophages, whereas free-living insects able to feed externally on plant tissues (e.g., leaf chewers, phloem suckers) were classified as exophages.

### Phylogenetic data

We constructed a phylogenetic hypothesis for all 582 recorded plant species (S4 Table) using a recently published [40] phylogeny of vascular plants, which includes over 30,000 species, belonging to 348 families and over 8,000 genera. This phylogeny was calibrated according to divergence time estimates from the broadly sampled molecular phylogeny of Soltis et al. [41] and using an additional 39 dated fossil calibration points [40]. Plant species in our compiled plant-herbivore assemblages that were absent from this phylogenetic tree (53% of the studied species) were included as polytomies at the node representing the most recent common ancestor of species from the same genus or family that were already present in the phylogeny. Out of the total of 272 species that we included, 239 were added at the genus level and the remaining 35 were included at the family level. The original tree was maintained ultrametric by assigning branch lengths of species as equal to the length between their point of insertion and the present (i.e., the tips representing species already present in the phylogeny). We then used this completed calibrated phylogeny to calculate the phylogenetic distance (in millions of years) among all pairs of plant species considered in the study. Finally, we computed for each host-plant species its mean phylogenetic distance from all native host species present in each plant-herbivore assemblage. This mean value was used as a measure of the phylogenetic isolation between each focal species and the co-occurring native species within the same local assemblage. Phylogenetic tree manipulation was performed in the R environment, version 3.1.0 [42], using original code and functions from the packages *adephylo* [43], *ape* [44], *geiger* [45], *phylobase* [46], *phytools* [47], and *picante* [48].

### Species richness and dissimilarity of insect herbivore faunas among plants

The herbivore species richness for each host plant species was obtained by counting the number of insect species associated with each of these plants. The species composition dissimilarity of the herbivore faunas between each focal host plant species and all other native species was calculated using the Jaccard and Simpson indices. The Jaccard dissimilarity index was calculated as (b + c)/(a + b + c), where “a” is the number of species found in both samples (i.e., both host species), and “b” and “c” were the number of species exclusively found in each one of the host plants. The Jaccard dissimilarity index accounts for differences in both species richness and species replacement [49]. The Simpson dissimilarity index was calculated as min(b,c)/min(b,c) + a, where “a”, “b”, and “c” were the same as for the Jaccard index. The Simpson index is not affected by differences in species richness, and thus only accounts for species replacement [49]. We then calculated the mean dissimilarity in herbivore species composition with the co-occurring native host plants for each host plant.

### Measuring host-plant specialization of herbivore insects

To assess the specialization of insect herbivores on their host-plant species for each plant-herbivore assemblage, we calculated the specialization index *d*’ of Blüthgen et al. [50]. This index is a standardized measure of the Kullback-Leibler distance, which measures the specialization of a species based on the frequency of the total number of interactions. The *d*’ index ranges from zero to one, where zero indicates maximum generalization and one indicates maximum specialization. We first calculated a global *d*’ for each insect herbivore species present in each plant-herbivore assemblage. We then calculated a mean *d*’ value for all insect herbivores associated with each host-plant species within an assemblage, thus obtaining a measure of the mean specialization of the herbivore fauna associated with each plant species. Specialization indices were calculated using the *bipartite* package [51] in R [42].

### Data analysis

We fitted a generalized linear mixed model (GLMM) for each of the response variables: herbivore richness, Simpson's and Jaccard's mean dissimilarities and the mean specialization of the herbivore fauna. In all of these models, phylogenetic isolation, host plant origin, and their interaction were considered as fixed effects, whereas the identity of each local assemblage was included as a random effect. In the model for herbivore richness, we also included the total herbivore richness of each assemblage as an offset term.

We used a Poisson error structure (count data) with the log-link function for the herbivore richness model, a Binomial error structure (proportion data) with the logit link function for the dissimilarity models, and a Gaussian error structure (continuous data) with the identity link function for the herbivore specialization model. In all models, we used a likelihood ratio test to determine whether to keep the interaction term, and removed it when the chi-squared value was not significantly different from zero. Conditional and Marginal R^2^ values were computed as an absolute measure of the goodness-of-fit of the models [52]. In some models, the identity of local assemblages (random affect) did not account for any variance, and in those cases, we refitted the models using a fixed-effects only GLMM and we used McFadden’s pseudo- R^2^ [53] as a measure of goodness-of-fit. All response variables and models were defined and fitted for each of the three assemblage datasets; the complete dataset considering all herbivores and those separately for the endophagous-only and exophagous-only plant-herbivore assemblages. Models were built using the *glmer* function in the *lme4* package [54] in R [42].

## Results

We recorded a total of 518 herbivorous species and 582 host plant species in the 30 plant-herbivore assemblages compiled. From these 30 assemblages, 17 were composed exclusively of exophages and 13, exclusively of endophages. The total number of insect herbivores associated with exotic and native host plants across all plant-herbivore assemblages was 177 and 429, respectively. The local species richness of insect herbivores within assemblages varied from five to 90 species (21.5 ± 23.8).

Since we did not find any significant interactions between the effects of plant phylogenetic isolation and plant origin (chi-squared values < 1.3, p < 0.25; Table 1), all GLMM models were subsequently fitted omitting the interaction term. This occurred for all three datasets, either including all assemblages or the endophagous and exophagous assemblages alone. Therefore, the effect of plant phylogenetic isolation on any of the response variables, when present, was the same for both native and exotic plants.

**Table 1:** Results of the likelihood ratio test to compare the models, either including or not including the interaction between phylogenetic isolation and plant origin, for the three different datasets (all herbivores, and assemblages of endophages or exophages) and the four models. P-values greater than 0.05 indicate no improvement in the models that include the interaction compared to those that do not include the interaction.

Assemblages	Response variable	Chi-squared	*P*
All herbivores	Species richness	0.141	0.708
	Jaccard dissimilarity	1.440	0.230
	Simpson dissimilarity	0.254	0.614
	Mean specialization of insect herbivores	0.297	0.586
Endophages	Species richness	1.058	0.304
	Jaccard dissimilarity	2.019	0.155
	Simpson dissimilarity	0.114	0.736
	Mean specialization of insect herbivores	< 0.001	0.992
Exophages	Species richness	0.131	0.717
	Jaccard dissimilarity	0.033	0.144
	Simpson dissimilarity	0.027	0.869
	Mean specialization of insect herbivores	1.55	0.213

**Fig 2:**
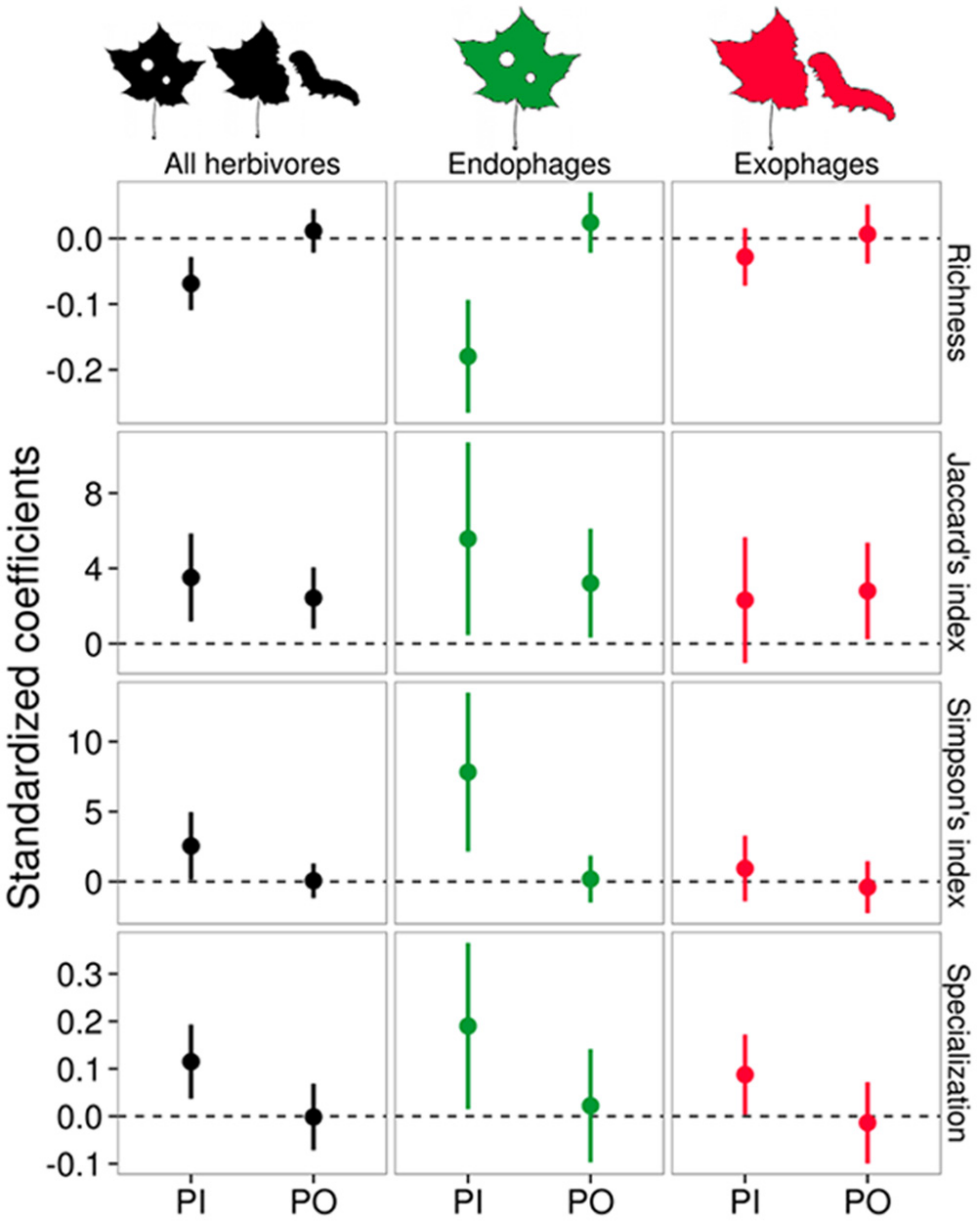
Standardized coefficients of the relationship between phylogenetic isolation (PI) and plant origin (PO) on the response variables (richness, Jaccard’s index, Simpson’s index and specialization) of insect herbivores, for all herbivores, only endophagous assemblages, or only exophagous assemblages. Positive coefficients for the plant origin effect mean higher values of the response variables on natives compared to exotic plants. Error bars represent ±1.96 standard errors.

Insect herbivore species richness on the host plants was negatively related to their phylogenetic isolation from other locally co-occurring plants (Table 2, Fig 2). For example, the herbivore richness on plants with the lowest mean phylogenetic isolation (5^th^ percentile) was 2.14 ± 1.55, whereas the herbivore richness on plants with the highest phylogenetic isolation (95^th^ percentile) was 1.45 ± 1.03. This effect of phylogenetic isolation on herbivore richness was observed using the complete dataset (z = -3.23, p < 0.001; Table 2) or for the assemblages composed exclusively of endophagous species (z = -4.09, p < 0.001; Table 2), but not for the exophagous insect assemblages (z = -1.42, p < 0.16; Table 2). In addition, there was no effect of plant origin on insect herbivore richness for any of the datasets (all herbivores: z = 0.67, p = 0.50; endophages: z = 1.03, p = 0.30 and exophages: z = 0.89, p = 0.38; Table 2).

**Table 2:** Generalized linear mixed model for the effects of phylogenetic isolation (PI) and plant origin (PO) on the richness of insect herbivores, for all herbivores, for assemblages of endophages only, or for assemblages of exophages only. Positive z-values for the plant-origin effects suggest a higher richness in native plants, whereas negative values suggest a higher richness in exotic plants. Marginal (R^2^_m_) and conditional (R^2^_c_) r-squared values are shown. The number of observations for the entire dataset includes 728 plant species in 30 local assemblages. The number of observations for the endophagous assemblages represents 215 plant species in 13 local assemblages, and that for the exophagous assemblages represents 513 plant species in 17 local assemblages.

	Fixed Effects				Random Effects		R^2^_glmm_	
Assemblages	PI		PO		Variance	SD	R^2^_m_	R^2^_c_
	z-value	*P*	z-value	*P*				
All herbivores	-3.323	**< 0.001**	0.674	0.500	0.653	0.808	0.017	0.598
Endophages	-4.094	**< 0.001**	1.031	0.303	0.816	0.903	0.122	0.707
Exophages	-1.418	0.156	0.886	0.376	0.090	0.300	0.008	0.165

We found high mean dissimilarity values between the composition of each focal plant species and their co-occurring native plants, with 75% of the Jaccard dissimilarity values greater than 0.84 and 75% of Simpson dissimilarity values greater than 0.67. Furthermore, the mean dissimilarity in the herbivore composition of focal plants increased with their phylogenetic isolation from their co-occurring plants (Table 3, Fig 2). This positive relationship was found for both the Jaccard and Simpson indices, in the complete and the endophagous datasets (Table 3), but not for the exophagous insect assemblages (Table 3). We also found an effect of plant origin on the mean Jaccard dissimilarity, with native plants showing higher mean dissimilarities than exotic plants in all datasets (all herbivores: z = 2.29, p = 0.02; endophages: z = 2.18, p = 0.03 and exophages: z = 2.14, p = 0.03; Table 3). Conversely, there was no effect of plant origin on the mean Simpson dissimilarities (all herbivores: z = 0.1 p = 0.92; endophages: z = 0.62, p = 0.83; exophages: z = -0.42, p = 0.67; Table 3).

**Table 3:** Generalized linear mixed model for the effects of phylogenetic isolation (PI) and plant origin (PO) on the mean dissimilarity of the herbivore fauna (Jaccard and Simpson dissimilarity indices), for all herbivores, only endophagous assemblages or only exophagous assemblages. Positive z-values for the plant-origin effects suggest a higher dissimilarity in the composition of insect herbivores in relation to native plants. Marginal (R^2^_m_) and conditional (R^2^_c_) r-squared values are shown, except for the models of Jaccard dissimilarity for the entire dataset and for exophagous insects, where we show McFadden’s pseudo- R^2^. The number of observations for the entire dataset include 728 plant species, in 30 local assemblages; that the endophagous assemblages represents 215 plant species in 13 local assemblages and that for the exophagous assemblages represents 513 plant species in 17 local assemblages.

Response variable	Assemblages	Fixed Effects				Random Effects		R^2^_glmm_	
		MPI		PO		Variance	SD	R^2^_m_	R^2^_c_
		z-value	*P*	z-value	*P*				
Jaccard dissimilarity	All herbivores	29.46	**0.003**	2.914	**0.004**	-	-	0.123	-
	Endophages	2.133	**0.033**	2.178	**0.029**	0.111	0.333	0.330	0.350
	Exophages	1.356	0.175	2.136	**0.033**	-	-	0.079	-
Simpson dissimilarity	All herbivores	2.064	**0.039**	0.099	0.921	4.068	2.017	0.060	0.580
	Endophages	2.699	**0.007**	0.616	0.833	9.327	3.054	0.360	0.830
	Exophages	0.782	0.434	-0.424	0.672	1.537	1.24	0.009	0.330

The mean specialization of herbivores on each host-plant was highly variable (0.56 ± 0.33), despite the high number of plants with a highly specialized fauna (25% of the plants had a mean specialization of their faunas equal to the maximum value of one). In all datasets (complete, endophagous-only, and exophagous-only assemblages), we detected an effect of host-plant phylogenetic isolation on the mean specialization of their associated herbivorous faunas, with more phylogenetically isolated host plants harboring more specialized insect herbivores (Table 4, Fig 2). However, we did not observe differences in mean herbivore specialization when comparing native and exotic plants in either of the datasets, (all herbivores: t = -0.04, p = 0.97; endophagous assemblages: t = 0.37, p = 0.72; endophagous assemblages: t = -0.82, p = 0.42; Table 4).

**Table 4:** Generalized mixed model for the effects of phylogenetic isolation (PI) and plant origin (PO) on the mean specialization of insect herbivores, for all herbivores, only endophagous assemblages or only exophagous assemblages. Positive t-values for the plant origin effects suggest a higher mean specialization of the herbivores on native plants, whereas negative values mean a higher suggest specialization on exotic plants. Marginal (R^2^_m_) and conditional (R^2^_c_) r-squared values are shown. The number of observations for the entire dataset represent 728 plant species, in 30 local assemblages; that for the endophagous assemblages represent 215 plant species in 13 local assemblages, and that for the exophagous assemblages represent 513 plant species in 17 local assemblages.

	Fixed Effects				Random Effects		R^2^_glmm_	
Assemblages	PI		PO		Variance	SD	R^2^_m_	R^2^_c_
	t-value	*P*	t-value	*P*				
All herbivores	2.883	**0.004**	-0.04	0.968	0.071	0.267	0.013	0.386
Endophages	2.127	**0.035**	0.365	0.715	0.034	0.185	0.041	0.278
Exophages	2.304	**0.021**	-0.815	0.415	1.239	1.113	0.021	0.289

## Discussion

Different ecological and evolutionary features of plants can influence the diversity of their insect herbivores. Here, we have shown that phylogenetic isolation of host plants is a key factor that influences the richness, composition, and specialization of their insect herbivore faunas. Our findings corroborate those of previous studies that found a negative correlation between host-plant phylogenetic isolation and insect herbivore richness [15,17], as well as at the herbivory level [55–56]. More importantly, we found no evidence that such effects of plant phylogenetic isolation on insect herbivore richness is different for plant species with different origins (native vs. exotic). Additionally, when controlling for phylogenetic isolation, we found no effect of plant origin on the mean composition dissimilarity of their insect herbivores in any of the assemblage datasets (except Jaccard dissimilarity index). This finding implies that, from the herbivore's point-of-view, the main source of difference between native and exotic host plants is their phylogenetic isolation from co-occurring plants. This also suggests that the factors that determine most aspects of plant-herbivore interactions in our studied assemblages are the same, regardless potential additional differences between native and exotic plants.

A key aspect of the plant-herbivore assemblages that was affected by the phylogenetic isolation of host plants was the richness of their insect herbivores. We found that the richness of insect herbivore species on host plants declined with an increase in the plants’ phylogenetic isolation. Phylogenetically isolated host plants, which are more distantly related to other co-occurring plants, are likely to possess novel and different traits within the assemblage that limit colonization by insect herbivores [57]. For example, chemical barriers might disrupt the olfactory recognition of those phylogenetically isolated host plants by the insect herbivores, probably due to unrecognized or avoided volatiles [26]. Such chemical barriers might gradually increase with the phylogenetic isolation of host plants, leading to a monotonic decrease in the richness of insect herbivores on more phylogenetically isolated host plants.

Our study also shows that the phylogenetic isolation of host plants is an important factor that determines the dissimilarity of their insect herbivores. In agreement with our initial prediction, we found that the composition dissimilarity of insect herbivores among plants tends to increase together with phylogenetic isolation of host plants. This finding corroborates that of previous studies. For example, Gossner et al. [27] found that exotic trees shared fewer insect herbivores with distantly related, native trees, concluding that the phylogenetic proximity of plants increases the similarity in the herbivore species composition among plants [27]. In fact, the species composition of insect herbivores associated with phylogenetically isolated plants might result from distinct evolutionary pools of insect herbivores. As a consequence, the compositional similarity between the insect herbivore faunas of closely and distantly related plants is less likely, because only a few insect herbivores can overcome the physical, chemical, and morphological barriers of distantly related plants. This effect acts in both directions, with the insect herbivores of phylogenetically isolated plants being unable to feed on other co-occurring plants, and insect herbivores of these plants being unable to colonize phylogenetically isolated plants.

The specialization of insect herbivores on their host plants was also dependent on the plant’s phylogenetic isolation. We found a positive relationship between the phylogenetic isolation of host plants and the specialization of their herbivore faunas. This finding contrasts with that of a recent study by Viallatte et al. [14], which showed that more phylogenetically isolated trees had herbivores that were less specialized [58–59]. Our own finding that insect herbivores on phylogenetically isolated plants are more specialized might be a consequence of these plants having insect pools that are evolutionarily distinct to those of the other co-occurring plants (see above). Indeed, specialized herbivorous insects would not be expected to incorporate novel plants that are phylogenetically distant from their original host species into their diets [60]. Furthermore, more specialized insects tend to be more effective at finding their hosts, which can in turn, limit their ability to recognize other plant species as potential hosts [61–62]. In addition, if natural enemies of insect herbivores are affected by the phylogenetic isolation of host plants, then the pressure exerted by natural enemies on insect herbivores might decrease [59], favoring a higher specialization of insect herbivores on phylogenetically isolated host plants. As a consequence, phylogenetically isolated host plants are more likely to harbor host-specialist herbivores than other locally co-occurring and more closely related plants.

As expected, the effect of the phylogenetic isolation of host plants on their herbivore faunas was dependent on the type of herbivory considered. Although we found significant relationships between host plant phylogenetic isolation and the richness and dissimilarity of their insect herbivores when considering all herbivores combined and endophages only, we did not find such relationships when only exophagous insects were considered. These results corroborate our expectation of a stronger effect of host-plant phylogenetic isolation on endophage diversity than on exophage diversity. Previous studies highlighted that exophagous herbivores are more tolerant to changes in their natural habitats, such as environmental changes due to land-use intensification and the increased dominance of exotic host plants [63]. Moreover, exophagous insect assemblages possess different feeding modes (e.g., adult chewers, larval chewers, phloem suckers) that and are composed of species with highly variable diets, ranging from very specialists to extremely generalist herbivores [5]. In fact, on average, exophagous species, have broader host ranges than endophagous species [5,32]. Additionally, the lower intimacy level of exophagous herbivores in relation to their hosts than endophages can allow these herbivores to include more phylogenetically distant plants in their diets.

Contrary to our expectation, we did not find evidence that exotic host-plant species support a lower herbivore richness and fewer specialist insects than co-occurring native plant species, when controlling for the effects of phylogenetic isolation. One explanation for this result might be that phylogenetic distance among plants becomes important only over a certain threshold. For instance, if there is a threshold for phylogenetic distance that limits the consumption of more phylogenetically isolated host plants by most herbivores, then it is plausible that phylogenetic isolation has a very limited effect on the variation in the number of herbivorous insects associated with exotic and native plant species. We included only host plant species that were consumed by at least one herbivore species; therefore the aim was not aim to investigate the effect of phylogenetic isolation on the likelihood of a given exotic or native plant species being attacked by at least one herbivore species. In addition, the absence of a difference in the host-plant specialization between herbivores associated with native and exotic plant species might be because the number of feeding specialist species is generally much higher than the number of feeding generalist species in most herbivore assemblages [29,31]. Therefore, although any particular generalist is expected to be more capable of consuming a given exotic plant, the likelihood of a given exotic plant being attacked by generalists and specialists will not be so different because specialists tend to be much more speciose than generalists. Similarly, we did not find an effect of phylogenetic isolation of host plants on the dissimilarity of insect herbivores (Simpson index) when native and exotic plants were compared. However, we did find that the Jaccard dissimilarity between focal host plants and the co-occurring natives was higher in native than exotic plants. This difference between the results of the Jaccard and Simpson indices could be explained by fact that herbivore richness has a higher weight in the calculation of the Jaccard's index, but we did not find evidence for this.

## Conclusions

This study highlights the importance of the phylogenetic isolation on the consumption of exotic and native host plants by herbivorous insects. In particular, we showed that phylogenetic isolation plays a significant role in determining the species richness, composition and specialization of insect herbivores associated with their host plants. Moreover, when controlling for the effects of phylogenetic isolation, the only detectable difference between native and exotic plants was in the dissimilarity of herbivore composition. This result shows that the plant origin influences herbivore occurrence at a finer scale, which is related to species identity and that determines the species composition, contrary to what was observed for richness and specialization of insect herbivores. Our findings have important practical implications; for instance, exotic host plants that are more phylogenetically isolated from native plants are more likely to escape from natural enemies in new areas [16]. As a consequence, such populations of phylogenetically isolated exotic plant species can grow faster, making their management more difficult. Future investigations are necessary to assess the effect of the phylogenetic isolation of host plants in the species richness, composition and specialization of their insect herbivores when considering other plant species that harbor no herbivorous insects, to detect potential thresholds of such effects on the host-plant use by insect herbivores.

## Supporting Information

S1 Table List of references of the 30 local plant-herbivore assemblages used in this study. (DOCX)

S2 Table Local characterization of the 30 plant-herbivore assemblages used in this study. References for the original studies are listed in S1 Table.
(DOCX)

S3 Table List of online database and sources used in the determination of exotic and native plant origin. (DOCX)

S4 Table List of 582 plant species recorded in the 30 local plant-herbivore assemblages. (XLSX)
