# Supplementary material for: The Effect of Host-Plant Phylogenetic Isolation on Species Richness, Composition and Specialization of Insect Herbivores: A Comparison between Native and Exotic Hosts
Source: PLoS One. 2015 Sep 17;10(9):e0138031. doi: 10.1371/journal.pone.0138031 (PMC4575019; doi:10.1371/journal.pone.0138031)
Supplement: S1 Table — (DOCX) [file pone.0138031.s001.docx]

Table S1. List of references of the 30 local plant-herbivore assemblages used in this study.

| Code | References |
| --- | --- |
| Borge and Basedow 1997 | Borge MNR, Basedow T (1997) A survey on the occurrence and flight periods of fruit fly species (Diptera: Tephritidae) in a fruit growing area in southwest Nicaragua. 1994/95. Bulletin of Entomological Research 87:405-412. |
| Cavalleri 2005 | Cavalleri A (2005) Comunidades de Tripes (Insecta: Thysanoptera) em flores e ramos, com ênfase em Asteraceae, no Parque Estadual de Itapuã, Viamão, RS. Master Thesis, Porto Alegre, Brazil. |
| Delfino et al. 2008 | Delfino MA, Buffa LM (2008) Afidos en plantas ornamentales de Córdoba, Argentina (Hemiptera: Aphididae). Neotropical Entomology 37:74-80. |
| Embrapa 2007 | Embrapa (2007) Avaliação ecológica de riscos de algodoeiro resistente a insetos: levantamento e seleção de lepidópteros não-alvo. Boletim de Pesquisa e Desenvolvimento 202:1-17. |
| Henneman and Memmot 2001 | Henneman ML, Memmot J (2001) Infiltration of a Hawaiian community by introduced biological control agents. Science 293:1314-1316. |
| Hernandez-Ortiz et al. 2006 | Hernandez-Ortiz V, Delfín-González H, Escalante-Tio A, Manrique-Saide P (2006) Hymenoptera parasitoids of *Anastrepha* fruit flies (Diptera: Tephritidae) reared from different hosts in Yukatan. Mexico. The Florida Entomologist 89:508-515. |
| Kollár 2011 | Kollár J (2011) Gall-inducing arthropods associated with ornamental woody plants in a City Park of Nitra (SW Slovakia). Acta entomologica serbica 16:115-126. |
| Leal 2008 (1,2) | Leal MR (2008) Dinâmica populacional das moscas-das-frutas (Diptera: Tephritidae) e introdução de *Diachasmimorpha longicaudata* Ashmead (Hymenoptera: Braconidae) para controle da praga na região norte do estado do Rio de Janeiro. Master Thesis, Seropédica, Brazil. |
| Leal 2009 | Leal RM, Souza SAS, Aguiar-Menezes EL, Lima-Fiolho M, Menezes EB (2009) Diversidade de moscas-das-frutas, suas plantas hospedeiras e seus parasitoides nas regiões Norte e Noroeste do Estado do Rio de Janeiro, Brasil. Ciência Rural 39:627-634. |
| Lopes and Basso 1974 | Lopes OJ, Link D, Basso LV (1974) Pentatomídeos de Santa Maria – lista preliminar de plantas hospedeiras. Revista Centro Ciências Rurais 4:317-322. |
| Mejías 2009 (1,2,3,4,5,6) | Mejías ZD (2009) Asociación de áfidos (Aphididae) en Costa Rica, su diversidad e interacciones tróficas con especies parasitoides (Braconidae: Aphidiinae) e hiperparasitoides (Hymenoptera). Tesis, Licenciatura en Ciencias Bilógicas con énfasis en Manejo integrado de plagas.Universidad de Costa Rica-Costa Rica. |
| Memmont et al. 1994 | Memmot J, Godfray HCJ, Gauld ID (1994) The structure of a tropical host-parasitoid community. Journal of Animal Ecology 63:521-540. |
| Peronti and Sousa-Silva 2002 | Peronti ALBG, Sousa-Silva CR (2002) Aphids (Hemiptera: Aphidoidea) of ornamental plants from São Carlos, São Paulo state, Brazil. Revista de Biología Tropical 50:137-144. |
| Perre et al. 2011 | Perre P, Loyola RD, Lewinsohn TM, Almeida-Neto M (2011) Insects on urban plants: contrasting the flower head feeding assemblages on native and exotic hosts. Urban Ecosystem 14:711-722. |
| Pinent et al. 2011 | Pinent SMJ, Romanowski HP, Redaelli LR, Cavalleri A (2011) Thysanoptera: plantas visitadas e hospedeiras no Parque Estadual de Itapuã, Viamão, RS. Brasil. Iheringia 95:9-16. |
| Starý and Havelka 2008 (1,2,3) | Starý P, Havelka J (2008) Fauna and associations of aphid parasitoids in an up-dated farmland area (Czech Republic). Bulletin of Insectology 61:251-276. |
| Tiple et al. 2011 | Tiple AD, Khurad AM, Dennis RLH (2011) Butterfly larval host plant use in a tropical urban context: Life history associations, herbivory, and landscape factors. Journal of Insect Science 11:65 available online: insectscience.org/11.65 |
| Uchôa et al. 2003 (1,2,3) | Uchôa-Fernandes MA, Molina RMS, Oliveira I, Zucchi RA, Canal NA, Diaz NB (2003) Larval endoparasitoids (Hymenoptera) of frugivorous flies (Diptera, Tephritoidea) reared from fruits of the cerrado of the State of Mato Grosso do Sul, Brazil. Revista Brasileira de Entomologia 47:181-186. |
| Uramoto et al. 2004 | Uramoto K, Walder JMM, Zucchi RA (2004) Biodiversidade de moscas-das-frutas do gênero *Anastrepha* (Diptera. Tephritidae) no *campus* da ESALQ-USP, Piracicaba, São Paulo. Revista Brasileira de Entomologia 48:409-414. |
| Uramoto et al. 2008 | Uramoto K, Martins DS, Zucchi RA (2008) Fruit flies (Diptera. Tephritidae) and their associations with native host plants in a remnant area of the highly endangered Atlantic Rain Forest in the State of Espírito Santo. Brazil. Bulletin of Entomological Research 98:457-466. |
|  |  |
