# Supplementary material for: The Effect of Host-Plant Phylogenetic Isolation on Species Richness, Composition and Specialization of Insect Herbivores: A Comparison between Native and Exotic Hosts
Source: PLoS One. 2015 Sep 17;10(9):e0138031. doi: 10.1371/journal.pone.0138031 (PMC4575019; doi:10.1371/journal.pone.0138031)
Supplement: S2 Table — References for the original studies are listed in S1 Table. (DOCX) [file pone.0138031.s002.docx]

Table S2. Local characterization of the 30 plant-herbivore assemblages used in this study. References of the assemblage original studies are listed in the Supplementary Material 1.

| Code | Country | Latitude | Longitude | Altitude  (m) |
| --- | --- | --- | --- | --- |
| Borge and Basedow 1997 | Nicaragua | 11 | -86 | 445 |
| Cavalleri 2005 | Brazil | -30.45 | -51.08 | 129 |
| Delfino et al. 2008 | Argentina | -37.872 | -64.784 | 474 |
| Embrapa 2007 | Brazil | -15.817 | -47.567 | 1075 |
| Henneman and Memmot 2001 | Hawaii | 22 | -159 | 771 |
| Hernandez-Ortiz et al. 2006 | Mexico | 20.3 | -89.7 | 38 |
| Kollár 2011 | Slovakia | 48 | 14 | 140 |
| Leal 2008 (1) | Brazil | -21.8 | -41.3 | 9 |
| Leal 2008 (2) | Brazil | -21.3 | -40.933 | 10 |
| Leal 2009 | Brazil | -21.3 | -40.933 | 10 |
| Lopes and Basso 1974 | Brazil | -29.683 | -53.783 | 131 |
| Mejías 2009 (1) | Costa Rica | 10.1306 | -84.2345 | 1430 |
| Mejías 2009 (2) | Costa Rica | 10.0779 | -84.5408 | 896 |
| Mejías 2009 (3) | Costa Rica | 9.8994 | -84.8788 | 18 |
| Mejías 2009 (4) | Costa Rica | 9.67878 | -84.8793 | 30 |
| Mejías 2009 (5) | Costa Rica | 9.93255 | -84.0464 | 1224 |
| Mejías 2009 (6) | Costa Rica | 9.50095 | -84.6136 | 85 |
| Memmont et al. 1994 | Costa Rica | 10 | -85 | 283 |
| Peronti and Sousa-Silva 2002 | Brazil | -22.015 | -47.891 | 830 |
| Perre et al. 2011 | Brazil | -22 | -47.069 | 876 |
| Pinent et al. 2011 | Brazil | -30.367 | -51.033 | 20 |
| Starý and Havelka 2008 (1) | Czech Republic | 49.513 | 14.931 | 537 |
| Starý and Havelka 2008 (2) | Czech Republic | 49.513 | 14.931 | 537 |
| Starý and Havelka 2008 (3) | Czech Republic | 49.513 | 14.931 | 537 |
| Tiple et al. 2011 | India | 21.65 | 80.65 | 319 |
| Uchôa et al. 2003 (1) | Brazil | -20.527 | -55.837 | 170 |
| Uchôa et al. 2003 (2) | Brazil | -20.65 | -55.331 | 173 |
| Uchôa et al. 2003 (3) | Brazil | -20.437 | -54.082 | 308 |
| Uramoto et al. 2004 | Brazil | -22.708 | -47.633 | 546 |
| Uramoto et al. 2008 | Brazil | -19.1 | -39.75 | 60 |
|  |  |  |  |  |
