# Supplementary material for: The Effect of Host-Plant Phylogenetic Isolation on Species Richness, Composition and Specialization of Insect Herbivores: A Comparison between Native and Exotic Hosts
Source: PLoS One. 2015 Sep 17;10(9):e0138031. doi: 10.1371/journal.pone.0138031 (PMC4575019; doi:10.1371/journal.pone.0138031)
Supplement: S3 Table — (DOCX) [file pone.0138031.s003.docx]

Table S3. List of online database and sources used in the determination of exotic and native plant origin.

| **Country** | **Online database or Source** | **Access date** |
| --- | --- | --- |
| Argentina | <http://www.floraargentina.edu.ar/espsin.asp> | 22-Apr-13 |
| Brazil | <http://floradobrasil.jbrj.gov.br/jabot/listaBrasil/PrincipalUC/PrincipalUC.do> | 17-Apr-13 |
| Costa Rica | <http://darnis.inbio.ac.cr/ubis/FMPro?-db=UBI&-lay=Weball&-format=findmore.html&-view> | 20-Apr-13 |
| Hawaii | <http://plants.usda.gov/du/DistributionUpdate.html> | 19-Apr-13 |
| Mexico | <http://www.conabio.gob.mx/invasoras/index.php/Especies_invasoras_-_Plantas> | 20-Apr-13 |
| Nicaragua | <http://www.tropicos.org/NameSearch.aspx?projectid=7> | 20-Apr-13 |
| Czech Republic | Pysek P, S adlo J and Mandák B (2003c) Alien ﬂora of the Czech Republic, its composition, structure and  history. In: Child LE, et al (eds) Plant Invasions: Biological Threats and Management Options, pp 113–130.  Backhuys Publishers, Leiden, The Netherlands | 21-Apr-13 |
| Czech Republic | Sadlo J, Chytrý M, & Pyšek P (2007) Regional species pools of vascular plants in habitats of the Czech  Republic. Preslia 79:303-321 | 21-Apr-13 |
| Czech Republic | Pyšek P, et al. (2012) Catalogue of alien plants of the Czech Republic (2nd edition): checklist update,  taxonomic diversity and invasion patterns. Preslia 84: 155–255 | 21-Apr-13 |
| India | Reddy CS (2008) Catalogue of invasive alien flora of India. Life Science Journal 5:84-89 | 9-May-13 |
| Mexico | Williams JK (2010) Additions to the alien vascular flora of Mexico, with comments on the shared species of Texas, Mexico, and Belize. Phytoneuron 3: 1-7 | 20-Apr-13 |
| Mexico | Villaseñor, J. L. y Espinosa-García, F.J. (2004). The alien flowering plants of Mexico. Diversity and Distributions 10: 113-12 | 20-Apr-13 |
| Slovakia | Jarolímek I. (2012): Inventory of the alien flora of Slovakia. Preslia 84: 257–309 | 21-Apr-13 |
